# Future Discounting in Congo Basin Hunter-Gatherers Declines with Socio-Economic Transitions

**DOI:** 10.1371/journal.pone.0137806

**Published:** 2015-09-18

**Authors:** Gul Deniz Salali, Andrea Bamberg Migliano

**Affiliations:** Department of Anthropology, University College London, London, United Kingdom; Institut Pluridisciplinaire Hubert Curien, FRANCE

## Abstract

Humans have a tendency to discount the future; that is we value small, short-term rewards over larger, long-term rewards. The degree of future discounting, however, changes in response to socio-ecological factors. Here, we study Mbendjele BaYaka hunter-gatherers of northern Congo and their farmer neighbours to investigate adaptations in inter-temporal preferences in humans. We argue that in immediate-return systems, where food storage is absent and egalitarianism is enforced through levelling mechanisms, future discounting is an adaptive strategy to prevent wealth accumulation and the emergence of hierarchies. This ensures food sharing and allows for survival in unpredictable environments where there is risk of an energy shortfall. On the other hand, when food storage is made possible by the emergence of agriculture or as seen in some delayed-return hunter-gatherer populations, wealth accumulation, hierarchies and lower discount rates become the adaptive strategy. Therefore, individuals in immediate-return, egalitarian societies will discount the future more than those in non-egalitarian, delayed-return societies. Consistent with the predictions we found that market integration and socio-economic transitions decrease the future discounting in Mbendjele hunter-gatherers. Our measures of socio-economic differences marked this transition in hunter-gatherers living in a logging town. The degree of future-discounting was the same between more market-integrated hunter-gatherers and their farmer neighbours.

## Introduction

Organisms often prefer immediate rewards over future ones, a preference that is known as “future discounting” [1]. A person with a higher discount rate (or higher impulsivity or lower patience) might choose 5 monetary units now, over 10 monetary units in a week. Although, the ability to fight impulsivity has been observed in non-human animals such as chimpanzees [2], pigeons [3] and guppies [4], humans appear to have evolved greater capacities for patience [5]. Some studies indicate that levels of future discounting are deep psychological attributes, linking early childhood time preferences with phenotypes developed later in life [6,7]. Others support the idea that choice of immediate versus delayed rewards varies more plastically in response to socio-ecological factors in both non-human primates [8,9] and humans [10,11].

Although there is a large body of literature investigating the determinants of future discounting in Western populations [7,11–14], there are only a handful of studies examining the level of future discounting in small-scale societies [15–21]. For example, Tucker [15] tested whether individual-level wealth and income variables or conformity to social norms better predicted time preferences in Mikea forest forager-farmers and their fisher and farmer neighbours. Godoy et al. [17] investigated whether the effects of years of schooling, age or income affected the levels of patience in Tsimane’ forager-horticulturalists. They also compared rates of discounting in Tsimane’ with those from two samples from the United States. While both studies found higher discount rates in forager-horticulturalist groups, they did not directly test whether a shift in social structure from an egalitarian one to a hierarchical one affects levels of future discounting. We believe investigating temporal preferences in foraging populations with egalitarian social structure may help us to understand when and how humans have developed greater patience.

Current-day hunter-gatherers present ideal populations to understand the trajectory of inter-temporal preferences in humans, since the decisions that extant hunter-gatherers make relative to their environment have a resemblance to the decisions that were made by pre-historic hunter-gatherers [22,23]. Some hunter-gatherer societies are immediate-return, where the obtained food is consumed in a short time. Immediate return hunter-gatherers are marked by high mobility and egalitarian political organization, which is believed to facilitate extensive food sharing as opposed to resource accumulation [24]. In delayed return systems (most farmers, herders and western systems) on the other hand, the food yield is obtained over long periods and stored; individuals accumulate wealth; and the political structure is less egalitarian and more hierarchical [24]. Immediate return societies sustain their egalitarian structure through levelling mechanisms that prohibit saving, accumulation and dominance [25–28]. These levelling mechanisms are believed to be important adaptations to ensure food sharing and in turn survival in unpredictable environments [27,29–31]. Transitions from immediate- to delayed-return systems are most likely triggered by new storage systems and domestication [23,32]. Changes in subsistence mode and food surpluses break down social systems of sharing, resulting in inequalities [29]. In a similar way, market integration, sedentism and high population density push immediate return hunter-gatherers towards a delayed-return system [23].

We suggest that future discounting in humans is a flexible behavioural adaptation associated with immediate-return systems and is subject to change in different socio-ecological conditions. The preference for immediate over delayed rewards will be adaptive in an immediate-return system where food is shared widely and consumed immediately without storage. In a society where individuals are obliged to share upon demand [33] and accumulation is prevented via norms, it will be risky to wait for larger rewards. With the dawn of agriculture and the possibility of food storage, which brought about the reduction of food sharing and the emergence of delayed-return economies, humans may have also developed delayed-return ways of thinking (i.e. patience) and adapted their behavioural strategies towards longer-term, higher-yield rewards. A shift away from immediate- towards a delayed-return system should therefore decrease individuals’ future discounting rates. Here we test whether: i) hunter-gatherers living in larger, more sedentary and market integrated groups will have more resource accumulation and less future discounting compared to mobile and forest-dwelling hunter-gatherers; ii) farmers will discount the future less than the hunter-gatherers. To test these predictions, we investigate the extent of future discounting and resource accumulation activities in Mbendjele BaYaka hunter-gatherers of the Republic of Congo who are characterized by their egalitarian social organization [34–37]. We compare inter-temporal decisions in hunter-gatherer camps living further away from a town with those who live in a town, and with Bantu farmers.

## Methods

### Study Population

There were 186 adult (> 15 years old) participants (91 females). Two Mbendjele camps (camp 1: *n* = 23, 14 females; and camp 2: *n* = 30, 18 females) were located in the forest, but close to mud roads opened by a logging company. In these camps subsistence was based on hunted and gathered products, although parts of hunted game were sold to bush meat traders. One Mbendjele camp (camp 3: *n* = 111, 46 females) was located in a logging town where most of the consumed products were bought in local markets. The last location was a Bantu farmer village (*n* = 22, 13 females; see S1 Supporting Information for further details).

Mbendjele BaYaka hunter-gatherers present an ideal population to study the adaptations in inter-temporal preferences in humans. They have an egalitarian social organization [35,37], however their way of life is changing because of increasing pressure from logging activities [38], commercial hunting, and protected forest areas for conservation [39,40]. Because camps vary in their proximity to logging towns, groups differ in their market integration and livelihood activities [39,40]. For example, during our field trip we observed frequent movement among hunter-gatherers living in the forest. Bush meat trade is common (the reason why many camps are located close to mud roads rather than deep in the forest), and sometimes individuals travel to the nearest farmer village to engage in wage labour [40]. Nevertheless, the main subsistence is hunting and gathering, and the money that is earned by selling meat is often spent immediately on goods such as alcohol, cigarettes, clothing and agricultural products. On the other hand, hunter-gatherers living in the logging towns engage in wage labour much more frequently [39], and they often buy their food from the local market with the money they earn via employment from farmers or the logging company. Individuals in forest camps use traditional round leaf and liana huts that can be easily abandoned and re-made to match their mobile lifestyle; whereas individuals living in towns have permanent houses made of old timbers (see S1 Supporting Information). In forest camps we observed very few personal possessions (such as pots, machetes, traditional baskets and mats, and some clothing) in households, however in town camps some houses even had furniture (e.g. old chairs) in them. Both the house type and large population densities (> 100 adults) indicate a transition to a sedentary lifestyle in town camps. It has been shown elsewhere that Mbendjele groups living in conservation-forestry towns spend much more time in formal employment, gun-hunting for villagers, wage labour and have become more sedentary [39].

### Experiment

The participants were informed that they were going to receive stock cubes (also known as bouillon cubes that are used widely in West and Central Africa as a food flavouring agent) for their participation in another study. Stock cubes are highly valued by Mbendjele to spice up food and obtained from traders, or local markets. At the end of the study, participants were asked whether they would prefer to receive one stock cube today or five tomorrow, and were given the stock cube(s) accordingly. This research was approved by UCL Ethics Committee, and carried out with permission from the Ministry of Scientific Research, Congo. Consent forms were verbally translated by a local translator, and questions from the participant were answered by researchers via the translator. Each participant, who agreed to take part, signed the informed consent form. The data supporting this article are available as part of the Supporting Information (S1 and S2 Datasets).

Data were analysed using R 3.2.0. To test whether people’s choices differed between locations, we performed logistic regression analyses. Our response variable was future discounting: the choice of one cube today was coded as 1; the choice of five cubes tomorrow was coded as 0. Our predictor variables were *Group type* (forest camps, town camp and Bantu village) and *Sex*. To obtain the optimal model, we removed non-significant variables based on the likelihood ratio test statistic and its associated p-value.

In the current study, we had two measures to examine the socio-economic differences between forest and town camps: 1-) sums paid as bride prices (*n* = 18 for forest camps, *n* = 26 for the town camp) as a proxy for resource accumulation, since bride price has to be accumulated and planned ahead [41]; 2-) frequency of farming work (*n* = 56 for forest camps, *n* = 63 for the town camp) as a proxy for integration in a delayed-return system [41]. We used a one-way permutation test based on Monte-Carlo simulations to compare the means of bride prices across locations. For the frequency of wage labour, we asked individuals how frequently they work for farmers, coding responses as 1) no/infrequent farm work, and 2) frequent farm work. We compared these responses using the Chi-square test of independence with Monte-Carlo re-samplings.

## Results

**Fig 1:**
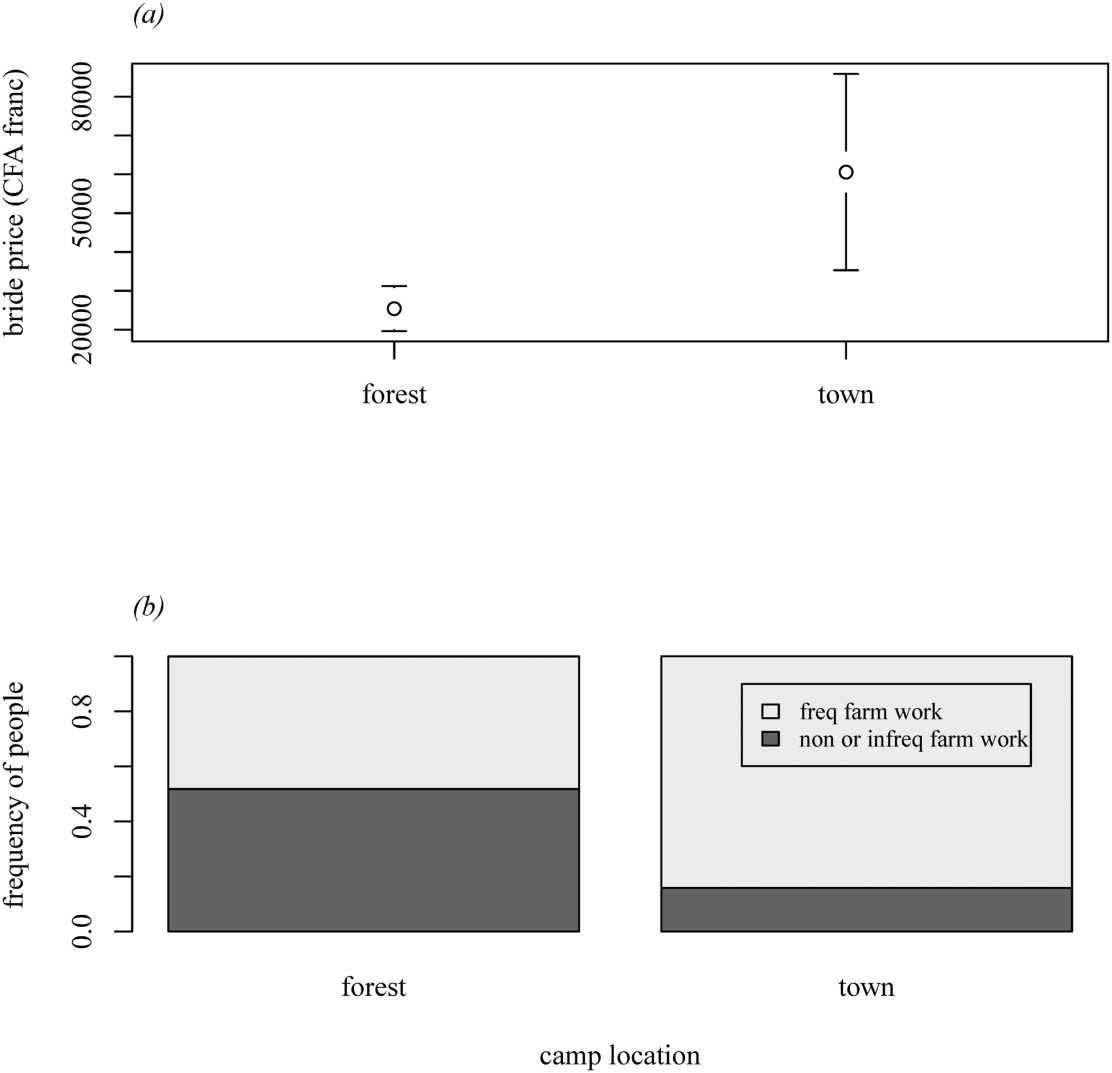
Socio-economic differences between hunter-gatherers living in forest camps and a town camp. *(a)* mean and 95% CI of the amount of bride price that men paid, *(b)* the frequency of wage labour in each location.

We first analysed the indicators of socio-economic differences between forest and town camps. These analyses revealed that hunter-gatherers in the town camp paid larger sums of bride price than those in forest camps (permutation test: *p* < 0.01; Fig 1a). The range of bride prices was larger in the town camp, indicating accumulation of resources and emerging inequalities (Fig 1a). Moreover, hunter-gatherers in the town camp engaged in wage labour much more frequently than those living in forest camps (*n* = 119, *χ* ^2^_1_ = 17.35, *p* < 0.001; Fig 1b).

**Fig 2:**
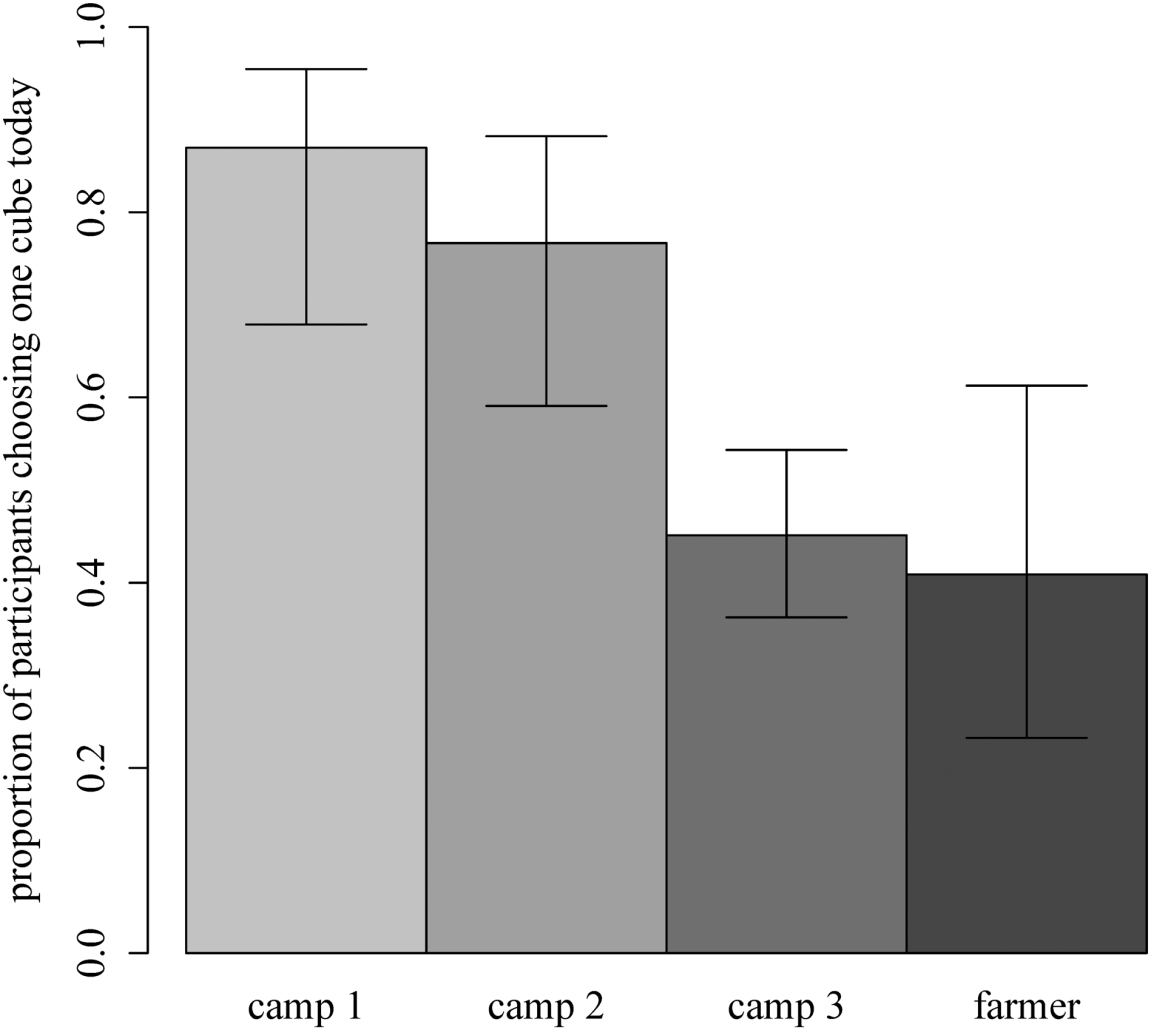
Proportion of participants that chose to receive one cube today in 3 Mbendjele camps and one Bantu farmer village. Camp 1 and camp 2 were located in the forest; camp 3 was located in the town. Error bars indicate 95% confidence intervals.

There was no difference between the proportions of participants that chose one cube today in the two hunter-gatherer forest camps (camp 1 and camp 2, *n* = 53, *χ* ^2^_1_ = 0.9, *p* = 0.48; Fig 2). Therefore, we pooled the data of camp 1 and camp 2 and refer to them as “forest camps” hereafter.

There was a significant difference in the probability of future discounting between the hunter-gatherers from the forest camps and the town camp (Fig 2). Table 1 shows our initial regression model with all the predictor variables (Model 1), and our optimal model (Model 2). According to Model 2, hunter-gatherers living in the forest discounted the future 5 times more than those in the town (odds ratio (OR) = 0.20, CI_.95_ = [0.09–0.43]). There was also a significant difference between hunter-gatherers living in the forest and Bantu farmers. Accordingly, odds of future discounting were 6 times higher in forest hunter-gatherers (OR = 0.16, CI_.95_ = [0.05–0.47]). Interestingly, there was no difference in future discounting between the town-living hunter-gatherers and farmers (*n* = 133, *χ* ^2^_1_ = 0.13, *p* = 0.82).

**Table 1:** Logistic regression models for probability of future discounting.

	Model 1		Model 2	
Predictor	Coeff. (SE)	P	Coeff. (SE)	P
Group type- town camp	-1.61 (0.41)	<0.001	-1.65 (0.40)	<0.001
Group type- Bantu	-1.83 (0.56)	<0.01	-1.83 (0.56)	<0.01
Sex- male	-0.30 (0.32)	0.43		
Intercept	1.58 (0.38)	<0.001	1.46 (0.35)	<0.001
Pseudo-*R* ^*2*^ (Hosmer-Lemeshow)	0.09		0.09	
-2 log likelihood	232.98		233.89	
*N*	186		186	

Response variable: future discounting (i.e. choosing one stock cube today over 5 stock cubes tomorrow). *Group type* encodes for the group of the participants (forest camps, town camp and Bantu agriculturalist village). *N* is the number of subjects. Omitting *Sex* did not affect the model fit significantly (*P*[*χ* ^2^(1) > 0.91] = 0.34).

For the subsample of the hunter-gatherers where we had information on frequency of wage labour and future discounting at the individual level, we found that those who engaged in frequent wage labour discounted the future less (Tables B-C in S1 Supporting Information). However, for the subsample of hunter-gather males (*N =* 38) the amount of bride price an individual paid did not have a significant effect on his future-discounting (Table D in S1 Supporting Information). In addition, our analysis on the effects of age on future discounting in Mbendjele showed no age effect (Tables E-H in S1 Supporting Information).

## Discussion

Consistent with our predictions, individuals in the town camp engage in more resource accumulation and have more variation in bride price payments, which is indicative of emergent inequalities. We show that immediate-return Mbendjele hunter-gatherers, that are less market integrated and engage in less resource accumulation activities, discount the future more than Mbendjele living in a logging town. Strikingly, those living in the town do not differ in their inter-temporal choices from Bantu farmers. Our results point to a group-level phenomenon; that socio-economic transitions affect the levels of future discounting; however we cannot specifically determine what factors play what role. It is possible that decreased mobility, increased population size, reduction in sharing and the change in subsistence mode, egalitarian norms and levelling mechanisms all contribute to the observed phenomenon in an intervening way. More individual-level measures are required to elucidate the effects of these factors.

The currency value matters in temporal choice experiments and resulting preferences may be study specific [15,17]. Although currency may matter, similar results in temporal discounting were found with different currencies, in different populations of foragers when compared to their neighbouring farmers, fishers or Western populations. For example, Mikea forest forager-farmers from Madagascar chose more immediate and low-risk options compared to their farmer and fisher neighbours [15]; and Tsimane’ forager-horticulturalists were on average more impatient compared to Western populations [17]. It requires further examination to establish whether the same decline in future discounting in the town camp would be observed by using a different currency (such as rice or money). Although discount rates may differ across different currencies, on average individuals living in a more delayed-return system will likely discount the future less regardless of the currency of choice.

An alternative interpretation is that time preference is a deep psychological attribute and not a flexible context-specific behavioural adaptation [17]. In this case the difference between forest camps and the logging town would be explained by assortment of individuals who are more future-oriented in logging towns [19]. If this is correct, then individual preferences would precede group effects. However, this interpretation does not explain the higher prevalence of preference for immediate rewards in forager societies compared to fisher, farmer or industrial populations found in this study, and others [15–17]. Thus these results suggest that the capacity for patience in humans is a plastic trait [42–44] that is expressed in response to socio-ecological conditions. Higher prevalence of patience in humans only became adaptive when food storage was possible. Nevertheless, an individual-level investigation analysing the change in time preferences in individuals moving from forest camps to towns may help us to further test behavioural flexibility in levels of patience.

Decisions on future discounting can also be analysed in light of life-history trade-offs [10]. According to life history theory, individuals adopt fast or slow reproductive strategies depending on resource availability and the stability of their environment [45], thus preference for immediate returns would be favoured in risky environments [46,47]. Accordingly, effects such as age and age specific mortality rates on life history strategies could also affect future discounting. In our study we did not find any effects of age estimates on inter-temporal preferences of the Mbendjele. Calculating mortality rates require adequate data on age estimates of both living and dead people (i.e. age at death and the year of birth) [48,49] and this data is not currently available for the Mbendjele. Therefore, it is not possible to confirm whether the mortality rates of the Mbendjele affect their levels of patience. Nevertheless, since hunter-gatherers live in unpredictable environments and thus depend more on immediate adaptive strategies such as fast life histories [50,51] and reliance on sharing rather than storage [30], it is expected that high mortality rates, and fast life history strategies may result in higher discount rates. More data is required to test these associations.

Our study corroborates the idea that in a society where sharing is enforced and accumulation is prevented via norms, it will be a risky strategy to wait for larger rewards. The sharing system, levelling mechanisms and future discounting can all be seen as social adaptations that ensure that immediate-return, egalitarian hunter-gatherers can buffer the pressures that result from unpredictable environments [29,30]. On the other hand, a change in the subsistence mode, possibility of food storage, sedentism, and increasing population size may all contribute to the emergence of wealth accumulation and inequalities [23]. As these changes occur humans may have adapted their behavioural strategies towards longer-term, higher-yield rewards. Nevertheless, there is only a handful of studies that focus on future-discounting in forager populations [15–17,21]. More comparative studies of immediate-return foragers versus delayed-return systems are necessary to shed light on the origins and flexibility of inter-temporal choices in humans.

## Supporting Information

S1 Supporting Information Detailed description of the methods and the results on the frequency of wage labour. (DOCX)

S1 Dataset Discount. Data on each participant’s discount choice (1 versus 5 stock cubes), sex, camp membership, age estimate, farming frequency and amount of bride price.
(XLSX)

S2 Dataset Socioeconomic indicators. Data on each participant’s amount of bride price paid, farming frequency, camp membership and sex.
(XLSX)
