# Supplementary material for: Future Discounting in Congo Basin Hunter-Gatherers Declines with Socio-Economic Transitions
Source: PLoS One. 2015 Sep 18;10(9):e0137806. doi: 10.1371/journal.pone.0137806 (PMC4575175; doi:10.1371/journal.pone.0137806)
Supplement: S1 Supporting Information — (DOCX) [file pone.0137806.s001.docx]

# Supporting Information

# Future discounting in Congo basin hunter-gatherers declines with socio-economic transitions

# Gul Deniz SALALI, Andrea B. MIGLIANO

Department of Anthropology, University College London, London, United Kingdom

## Methods

Mbendjele BaYaka hunter-gatherers (who are also known as one of the many groups of Central African Pygmies [1]) live in the equatorial forests of the Northern Republic of Congo, where the fieldwork took place between April and August 2014. The two Mbendjele camps (camp 1 and camp 2) were located in the forest (Fig. S1a), close to the mud roads that were opened by a logging company. One Mbendjele camp (camp 3) was located in a logging town (Fig. S1b). Fig. S2 shows the locations of the Mbendjele camps and a Bantu farmer village and table S1 shows the distances from the logging town for each Mbendjele camp.

We spent equal amounts of time, 3-4 weeks, in each camp regardless of whether they were located in the forest or in the town. In the forest camps, we established our camp right next to the Mbendjele camp, and were actively participating in the daily/social activities such as foraging for various forest products and evening dances. Participants were aware that we were staying there not for a day, but a longer period of time. Moreover, we performed this study after trust had been built and the groups were familiar with us.

a)


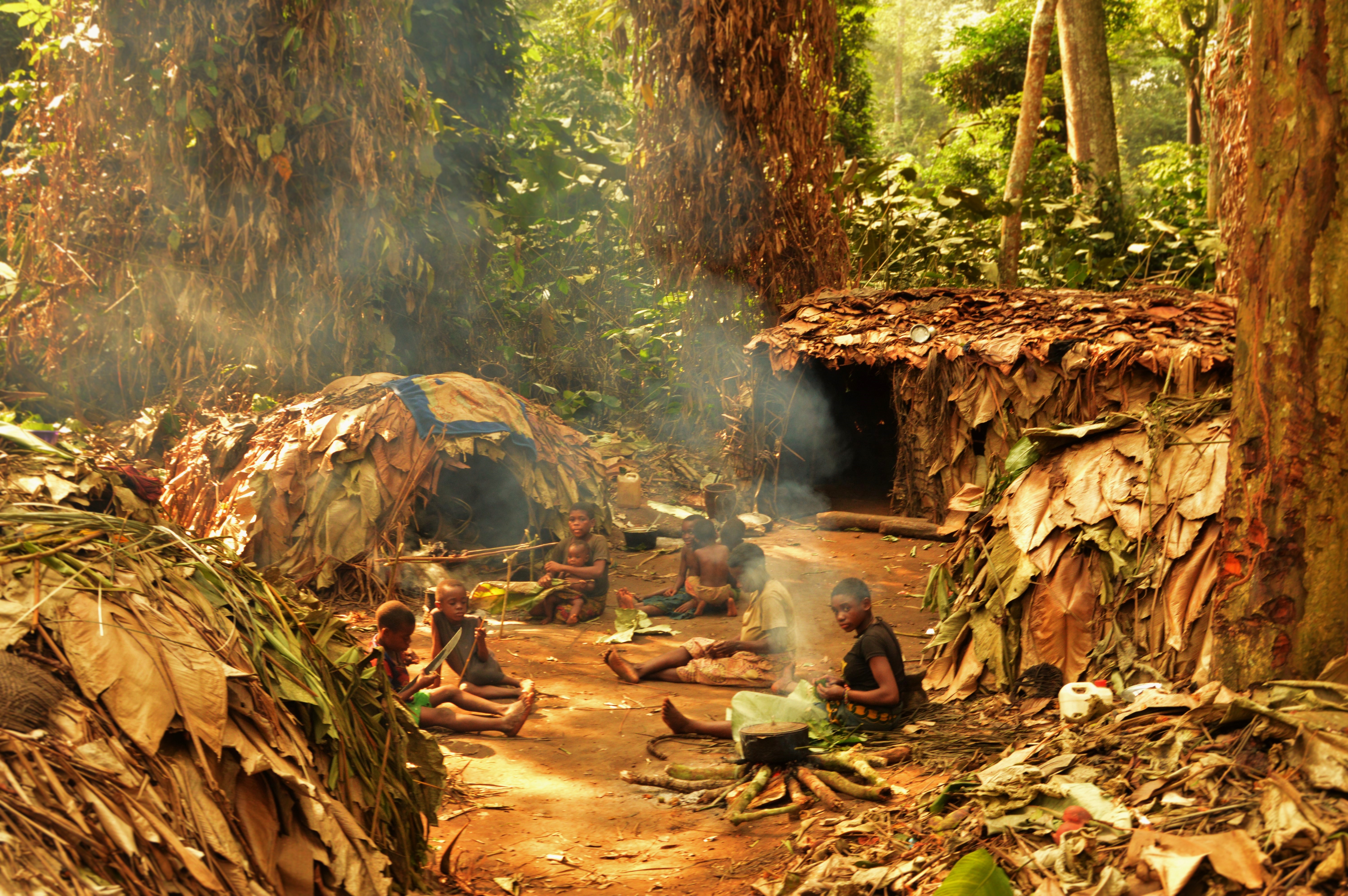


b)


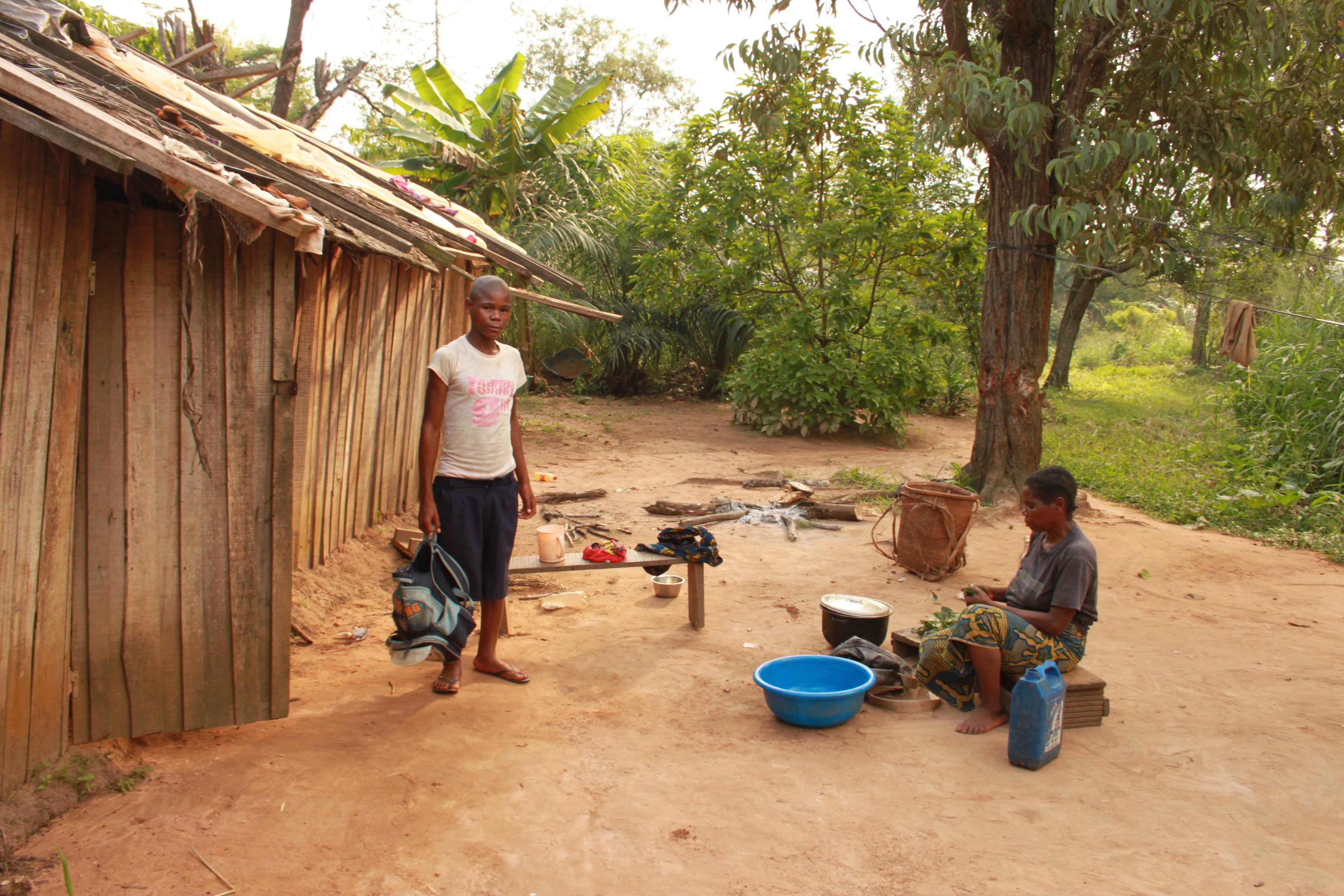


Fig. A. 1) A scene from the Mbendjele forest camp 1 (photo credit: Nikhil Chaudhary), 2) A Mbendjele family and their house in the town camp (photo credit: Gul Deniz Salali).


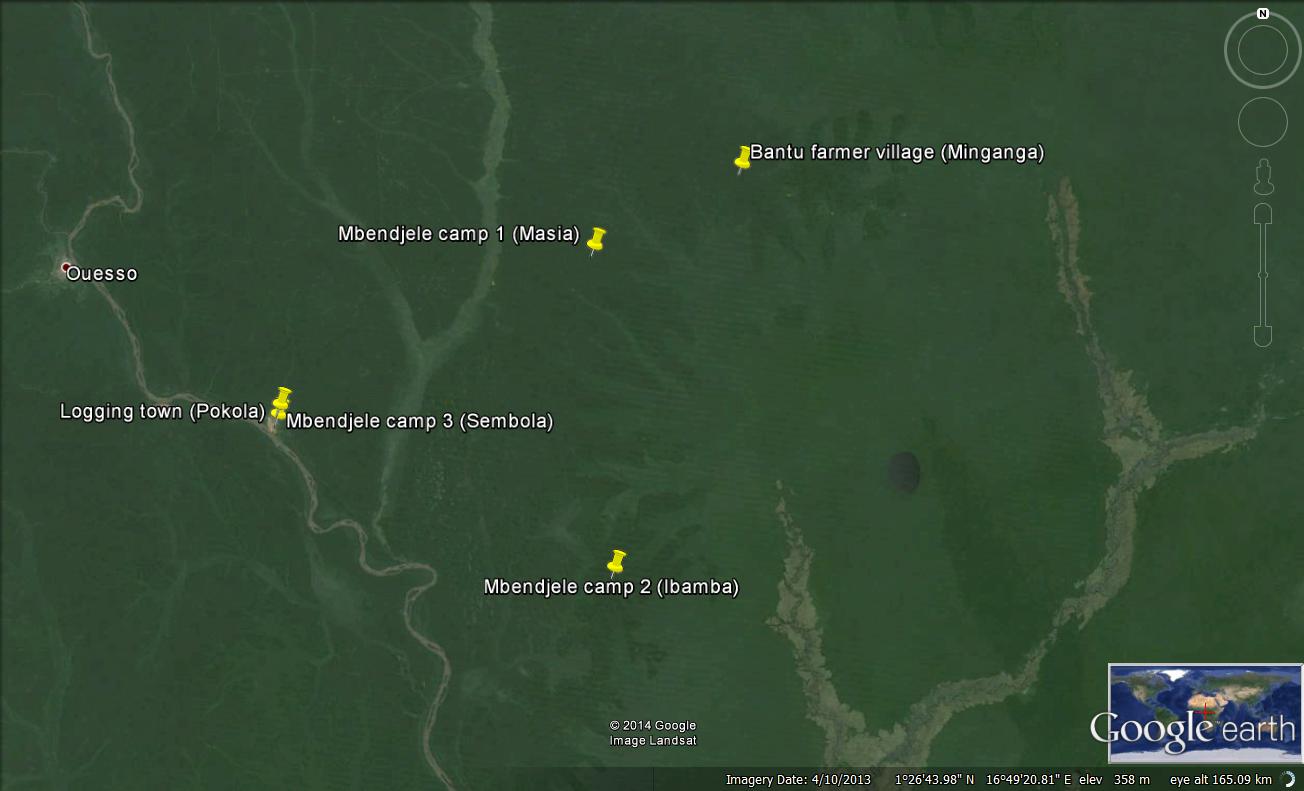


Fig. B. Location of the main research sites (marked by yellow pins)

Table A

Distance from the market town for each Mbendjele camp

| Location | Distance from the nearest market town* |
| --- | --- |
| Mbendjele forest camp 1 | 60 km |
| Mbendjele forest camp 2 | 100 km |
| Mbendjele town camp 3 | 0 |

*Distance is calculated based on the mud roads that were used to travel between the two GPS coordinates.

We designed a choice task between receiving one stock cube today versus 5 stock cubes tomorrow because our pilot experiments conducted in a forest camp with a larger temporal gap and varying amounts of reward (e.g. one stock cube tomorrow or 10 stock cubes in 5 days) resulted in no variation, where all participants discounted the future. In addition, most Mbendjele do not have experience in counting large numbers, therefore we kept our study design as simple as possible.

## Frequency of wage labour and future discounting

We used two indicators (amount of bride price and frequency of engagement in wage labour) to quantitatively demonstrate the proposed transition from an immediate to a delayed-return system amongst Mbendejele living in the town. However, other factors such as the level of food sharing, the proportion of food that is obtained from the market versus that is hunted or gathered, the frequency of movement between camps may all reflect this transition. Since the transition is a group-level phenomenon that results from various intervening factors, we used group type (forest, town or farmer) as a predictor in our main analysis.

For a subsample of our study population, we had information on both the frequency of wage labour in farms and the future discounting at the individual level (*n* = 100, 55 females). To test whether farming frequency (coded as either “non/infrequent” or “frequent”) predicts the levels of future discounting we performed a separate logistic regression analysis, controlling for the sex of the participants. The optimal model obtained from this analysis showed that individuals who engaged in frequent wage labour in farms discounted the future about 3 times less than the individuals who never or only infrequently engaged in wage labour (OR = 0.35, CI_.95_ = [0.13-0.88]; table S2).

Table B

Logistic regression models for the probability of future discounting with frequency of wage labour as a predictor

|  | Model 1 | | Model 2 |  |
| --- | --- | --- | --- | --- |
| Predictor | Coeff.  (SE) | P | Coeff.  (SE) | P |
| Wage labour frequency- frequent | -1.05  (0.49) | <0.05 | -1.04  (0.48) | <0.05 |
| Sex – male | -0.61  (0.43) | 0.16 |  |  |
| Intercept | 1.39  (0.48) | <0.01 | 1.10  (0.41) | <0.01 |
| Pseudo-*R^2^* (Hosmer-Lemeshow) | 0.05 |  |  | 0.04 |
| -2 log likelihood | 128.10 |  |  | 130.20 |
| *N* | 100 |  |  | 100 |

Logistic regression coefficients, standard errors and P-values. Response variable: future discounting (i.e. choosing one stock cube today over getting 5 stock cubes tomorrow). *Wage labour frequency* encodes for the frequency of wage labour (non/infrequent or frequent wage labour). *N* is the number of subjects. We used quasi-binomial logistic regression when the residual deviance exceeded the residual degrees of freedom. Omitting *Sex* did not affect the model fit significantly (*P*[*χ*^2^(1) > 2.10] = 0.15).

Finally, to test whether the group type remains a significant predictor after controlling for the frequency of wage labour we performed the above analysis by adding the group type as one of the main predictors. It should be noted that in this analysis, group type included forest camps and town camp, but not the farmer village since we only collected data on frequency of wage labour from the Mbendjele. The model revealed group type as the only significant predictor for future discounting. Accordingly, odds of future discounting were 5.5 times higher in forest hunter-gatherers compared to the ones living in the town (OR = 0.18, CI_.95_ = [0.06-0.48]; table S3). The fact that frequency of wage labour lost its significance suggests that the wage labour effects were actually the result of the group type. This is not a surprising result, as wage labour is only one of the factors involved in the transition from an immediate to a delayed-return system. In order to be able to capture the whole effect of group type we probably would have to have individual measures of food sharing, mobility, trade, wage labour, consumption of agricultural products, frequency of hunting trips etc., since the logging town and the forest camps have very different lifestyles.

Table C

Logistic regression models for the probability of future discounting with frequency of wage labour and group type as the main predictors

|  | Model 1 | |
| --- | --- | --- |
| Predictor | Coeff.  (SE) | P |
| Group type- town camp | -1.72  (0.53) | <0.01 |
| Wage labour frequency- frequent | -1.05  (0.49) | 0.53 |
| Sex – male | -0.61  (0.43) | 0.22 |
| Intercept | 1.39  (0.48) | <0.001 |
| Pseudo-*R^2^* (Hosmer-Lemeshow) | 0.15 |  |
| -2 log likelihood | 115.55 |  |
| *N* | 100 |  |

## Bride price and future discounting

We have conducted additional analyses to test the effect of amount of bride price paid by males on future discounting. Since the information on bride prices was only available for married men, we had data on the amount of bride prices for 18 males in the forest camps, and 26 males in the town camp. Among those 44 males we had data for 38 of them on their future discounting. Our logistic regression analysis showed that the amount of bride price paid by a male did not affect his choice in the discount experiment, in this subset of the sample (see the table below).

Table D

Logistic regression models for the probability of future discounting with the amount of bride price paid as a predictor

|  | Model 1 | | | Model 2 | | |
| --- | --- | --- | --- | --- | --- | --- |
|  | Coeff | SE | P | Coeff | SE | P |
| Bride price (amount) | 0 | 0 | 0.16 | 0 | 0 | 0.39 |
| Camp- town |  |  |  | -1.51 | 0.85 | 0.08 |
| (Intercept) | 0.67 | 0.54 | 0.23 | 1.41 | 0.72 | 0.06 |
| N | 38 |  |  | 38 |  |  |
| Pseudo-*R^2^* (Hosmer-Lemeshow) | 0.06 |  |  | 0.13 |  |  |
| -2 log likelihood | 49.34 |  |  | 45.64 |  |  |

## Age effects on future discounting in Mbendjele

In our sample, we had consensus relative age lists (a method explained in detail by [2]) for the two forest camps. We used these two age ranks to break the data into three age categories: young adult, adult and old adult. Since we did not have a relative age list for the town camp, we used eyeball estimates of ages that were recorded during data collection. Accordingly, we listed any participant that looked between 15-25 as young adults, others older than 25 and younger than 45 as adults, and everyone else (45+) as old adults. We did not have any recording for the Bantu farmers, so in our supplementary analysis for age we excluded the farmers. Table S5 shows groups of participants by camp.

Table E

Age groups by camp

| Camp | Young adult | Adult | Old adult | NA |
| --- | --- | --- | --- | --- |
| Forest camp 2 | 4 | 15 | 11 | 0 |
| Forest camp 1 | 5 | 14 | 4 | 0 |
| Town camp | 28 | 54 | 26 | 3 |

We ran logistic regression analysis to test whether the age group of a participant affected his/her choice. Our analysis showed no effect of age group on future discounting (Table S6).

Table F

Logistic regression model for the probability of future discounting with age group, group type and sex as the main predictors

| **Predictor** | **Coeff.** | **SE** | **P** |
| --- | --- | --- | --- |
| Sex- male | -0.58 | 0.36 | 0.11 |
| Group type- forest camp 1 | 0.73 | 0.77 | 0.35 |
| Group type- town camp | -1.27 | 0.49 | 0.01 |
| Age group- adult | -0.23 | 0.44 | 0.6 |
| Age group- old adult | -0.04 | 0.5 | 0.94 |
| (Intercept) | 1.58 | 0.59 | 0.01 |
| Pseudo-*R^2^* (Hosmer-Lemeshow) | 0.11 |  |  |
| -2 log likelihood | 195.98 |  |  |
| N | 161 |  |  |

For age group, the baseline is young adult.

Since the relative age list is a better estimate for the age of participants, we also ran logistic regressions using age ranks of participants as a continuous response variable for the two forest camps. In both analyses age ranks did not influence participants’ inter-temporal choices (Table S7).

Table G

Logistic regression model for the probability of future discounting with age ranks and sex as the main predictors

|  | Model- forest camp 2 | | | Model- forest camp 1 | | |
| --- | --- | --- | --- | --- | --- | --- |
| Predictors | Coeff | SE | P | Coeff | SE | P |
| Sex- male | -0.36 | 0.94 | 0.7 | -1.32 | 1.36 | 0.33 |
| Age rank | 0.05 | 0.05 | 0.34 | 0 | 0.02 | 0.8 |
| (Intercept) | -0.55 | 2.15 | 0.8 | 1.69 | 3.26 | 0.6 |
| Pseudo-*R^2^* (Hosmer-Lemeshow) | 0.04 |  |  | 0.06 |  |  |
| -2 log likelihood | 27.98 |  |  | 16.52 |  |  |
| N | 28 |  |  | 22 |  |  |

Because in the above analyses each camp represents a small sample with little variation in the response variable, we did an additional analysis where we calculated age estimates for the people in the two forest camps. We did this by using the age ranks and anchoring points where some individuals’ ages were known. These anchor points were obtained by asking the participants whether the birth of an individual coincided with key events in the past with a known date, such as establishment of a logging road. In the next analyses we used these estimated ages and combined the responses of the two forest camps. Again, age did not have an effect on future discounting.

Table H

Logistic regression model for the probability of future discounting with age estimates for the forest camps and sex as the main predictors

| Predictor | Coeff | SE | P |
| --- | --- | --- | --- |
| Sex-male | -0.38 | 0.8 | 0.64 |
| Age estimate | 0 | 0.02 | 0.89 |
| (Intercept) | 1.52 | 1.1 | 0.18 |
| Pseudo-*R^2^* (Hosmer-Lemeshow) | 0.01 |  |  |
| -2 log likelihood | 42.40 |  |  |
| N | 40 |  |  |

## References

1. Lewis, J. 2014 Egalitarian social organization: the case of the Mbendjele BaYaka. In *Hunter-Gatherers of the Congo Basin* (ed B. S. Hewlett), pp. 219–243. New Brunswick (U.S.A) and London (U.K): Transaction Publishers.

2. Hill, K. & Hurtado, A. M. 1996 *Ache Life History: The Ecology and Demography of a Foraging People.* Aldine Press.
